# Profile of gonadotropin secretion in male and female rats determined in the tail‐tip blood by ultrasensitive ELISA

**DOI:** 10.1111/jne.70157

**Published:** 2026-03-10

**Authors:** Roberta Araujo‐Lopes, Andre F. Gomes, Matheus Viana, Mariana De S. Santos, Ana C. Campideli‐Santana, Soraia Macari, Adelina M. Reis, Raphael E. Szawka

**Affiliations:** ^1^ Departamento de Fisiologia e Biofisica Instituto de Ciencias Biologicas, Universidade Federal de Minas Gerais Belo Horizonte Brazil; ^2^ Departamento de Odontologia Restauradora, Faculdade de Odontologia Universidade Federal de Minas Gerais Belo Horizonte Brazil

**Keywords:** enzyme‐linked immunosorbent assay, estrous cycle, follicle‐stimulating hormone, gonadectomy, luteinizing hormone

## Abstract

There is a need for accurate and less invasive methods of hormonal measurements. Here, we longitudinally determined luteinizing hormone (LH) and follicle‐stimulating hormone (FSH) secretion measured in the tail‐tip blood of male and female rats by ultrasensitive enzyme‐linked immunosorbent assay (ELISA). In males, the ELISAs detected a significant increase in LH and FSH secretion after orchiectomy compared with the gonad‐intact condition. The subsequent treatment with testosterone in the orchiectomized condition returned LH concentrations to the basal levels, whereas FSH secretion was partially restored to the gonad‐intact levels. During the rat estrous cycle, LH and FSH secretion fluctuated around basal levels on diestrus, and the preovulatory surges of both hormones occurred on the late afternoon of proestrus. On estrus, LH secretion was continually low, while FSH concentrations progressively declined from elevated concentrations in the morning to lower levels in the afternoon. After ovariectomy, secretion of both LH and FSH rose above the basal levels of the estrous cycle. Estradiol treatment in the ovariectomized condition reduced LH and FSH secretion towards gonad‐intact levels in the morning, with a greater effect on FSH. In the afternoon, estradiol treatment prompted a sharp proestrus‐like surge of LH alongside a slower, gradual increase in FSH levels. In the present study, we characterize LH and FSH secretion in rats of both sexes under different hormonal conditions, using longitudinal hormonal measurement in the tail‐tip blood by ultrasensitive ELISA. These findings provide novel methodological and conceptual information about gonadotropin secretion in rats relevant to the knowledge in reproductive endocrinology.

## 1 INTRODUCTION

The coordinated release of the gonadotropins is essential for the appropriate control of the hypothalamus‐pituitary‐gonadal (HPG) axis and fertility in males and females, driving gametogenesis and the secretion of gonadal hormones.^1^, ^2^ Luteinizing hormone (LH) and follicle‐stimulating hormone (FSH) are secreted by the anterior pituitary gland in a net response to the stimulation by the hypothalamic gonadotropin‐releasing hormone (GnRH) and the feedback action of gonadal steroids. Estradiol in females and testosterone in males exert negative‐feedback effects on the HPG axis, reducing LH and FSH secretion.^3^, ^4^, ^5^, ^6^, ^7^ In addition, in different species of spontaneously ovulating females, such as rodents and primates, a sustained rise in circulating estradiol levels elicits the positive‐feedback effect to activate GnRH neurons and thereby trigger the preovulatory LH surge and ovulation.^8^, ^9^, ^10^, ^11^ In rodents, this mechanism is exclusive to females, whereas males do not display an LH surge even when treated with high doses of estradiol after castration.^12^, ^13^

The measurement of gonadotropin secretion is clinically relevant in assessing reproductive function. For instance, FSH and LH levels are routinely evaluated in the diagnosis of precocious puberty, disorders of sexual development, hypogonadotropic hypogonadism, infertility in both sexes, and menopause.^14^, ^15^, ^16^, ^17^ Accordingly, LH and FSH measurements have long been performed by radioimmunoassay (RIA) in several species.^18^, ^19^, ^20^, ^21^, ^22^, ^23^ In male rats, RIA has been used to accurately determine physiological changes in gonadotropin secretion, including the effects of castration and testosterone replacement on LH and FSH levels.^24^, ^25^ The secretory profile of LH and FSH throughout the estrous cycle of female rodents has also been characterized by RIA,^10^, ^26^, ^27^ and the preovulatory LH surge of proestrus was first demonstrated using this method.^28^, ^29^ RIA has also been effective in detecting the increase in LH and FSH levels after ovariectomy and the reduction in both hormones in estradiol‐treated ovariectomized (OVX) rats.^30^, ^31^ However, although reliable, RIA depends on radioisotopes and generally requires a considerable volume of plasma or serum for the analysis. These features limit its potential as a widespread method of hormonal measurement and are hardly compatible with studies that require repeated hormonal measurement over time. Therefore, there is great interest in developing new non‐invasive methodologies capable of quantifying hormone levels in tiny volumes of blood in a reliable, accurate, and sensitive manner.

Of note, ultrasensitive enzyme‐linked immunosorbent assays (ELISAs) have been developed to measure growth hormone, LH, prolactin, and FSH in the whole blood collected from the tail tip of mice.^32^, ^33^, ^34^, ^35^ The ultrasensitive LH and prolactin ELISAs have been later adapted to the hormonal measurement in the tail‐tip blood of rats.^36^, ^37^, ^38^ Notably, this methodology requires only minimal blood volume for the assay, allowing for serial and repeated blood sampling over time without the adverse effects of significant blood loss or stress caused by the surgeries for blood‐vein cannulation. Although an ultrasensitive FSH ELISA has been developed for use in mice,^33^ a similar assay has not been tested in rats. More importantly, the physiological profile of gonadotropin secretion in rats, as measured in the tail‐tip blood by ELISA, is to be determined.

In the present study, we tested an in‐house ultrasensitive FSH ELISA for rats and used this ELISA methodology to longitudinally characterize LH and FSH secretion in the tail‐tip blood of male and female rats under different hormonal conditions. The findings reported here demonstrate the effectiveness of these assays in measuring gonadotropin secretion in rats and further characterize the variations in LH and FSH levels during the estrous cycle, the negative‐feedback regulation by gonadal steroids in males and females, and the positive‐feedback effect of estradiol on LH and FSH secretion in OVX rats.

## 2 MATERIALS AND METHODS

### 2.1 Animals

Male and female Wistar rats weighing 250 to 300 g were separated by sex, group housed in 4 per cage under conditions of controlled lighting (lights on 7:00 to 19:00 h) and temperature (22 ± 2°C), and provided with standard laboratory chow and water ad libitum. Vaginal smears were taken daily, and rats showing at least three consecutive regular estrous cycles^39^ were included in the experiments. All procedures were approved by the Ethics Committee on the Use of Experimental Animals of the Universidade Federal de Minas Gerais (CEUA‐UFMG 216/2022).

### 2.2 Experimental design

#### 2.2.1 Experiment 1: Validation of rat LH and FSH ultrasensitive ELISAs

LH and FSH ultrasensitive ELISAs have been developed for use in mouse,^32^, ^33^ and we have previously used ultrasensitive ELISAs for measuring LH and PRL in rat blood.^36^, ^37^ As the FSH ELISA has not been validated for FSH measurement in rats, here we tested a modified FSH ELISA protocol to be used in this species. This experiment was performed to validate the effectiveness of the LH and FSH ELISAs for accurate hormonal detection in rats. Initially, we tested whether the rat plasma would interfere with the immunoreactivity of the assays. Subsequently, we determined the efficacy of the ELISAs in detecting changes in LH and FSH concentrations in gonad‐intact and ovariectomized (OVX) rats. Cycling rats with 3 months of age were OVX or sham‐operated and were euthanized after 1 (Sham‐1 M; *n* = 7; OVX‐1 M; *n* = 8), 2 (Sham‐2 M; *n* = 7; OVX‐2 M; *n* = 8), or 6 (Sham‐6 M; *n* = 8; OVX‐6 M; *n* = 8) months. Trunk blood was collected after decapitation, and plasma LH and FSH levels were measured by the ultrasensitive ELISAs.

#### 2.2.2 Experiment 2: Effect of orchiectomy and testosterone replacement on LH and FSH secretion in rats determined by ultrasensitive ELISA

Orchiectomy increases LH and FSH secretion due to the removal of testosterone and inhibin negative‐feedback effects at the hypothalamus and pituitary.^40^, ^41^, ^42^ We aimed to determine the changes in LH and FSH secretion after orchiectomy and testosterone replacement in male rats, measured in the tail‐tip blood by ultrasensitive ELISA. We employed a longitudinal approach in which the same rats were sequentially evaluated in three different hormonal conditions, thereby serving as their own controls (*n* = 6). Three tail‐tip blood samples of 20 μL were withdrawn hourly from 10:00 to 12:00 h in each experimental condition. The measurements of the three blood samples were averaged per rat for each experimental condition. Initially, gonad‐intact rats underwent the tail‐tip blood collection (Intact condition). Within 5 days, rats were orchiectomized (ORX) and, after 10 days of recovery, received subcutaneous (s.c.) treatment with corn oil for 7 consecutive days, and the blood collection was performed on the following day (ORX condition). Fifteen days later, the ORX rats were treated with testosterone for 7 consecutive days, and the protocol of tail‐tip blood sampling was performed on the following day (ORX + T condition). LH and FSH levels were measured in the whole blood by the ultrasensitive ELISAs.

#### 2.2.3 Experiment 3: LH and FSH secretion during the rat estrous cycle determined by ultrasensitive ELISA

The patterns of LH and FSH secretion during the rat estrous cycle have been established essentially using terminal blood collection and plasma RIA.^10^, ^26^, ^28^ This experiment aimed to further characterize the temporal profiles of LH and FSH secretion during the rat estrous cycle, using serial whole blood samples measured by ELISA. The estrous cycle days were defined according to the characteristic cell types in the vaginal cytology.^39^ Ten tail‐tip blood samples of 20 μL were collected hourly from 10:00 to 19:00 h from different cohorts of rats on diestrus (diestrus 2; *n* = 7), proestrus (*n* = 6), or estrus (*n* = 6). LH and FSH were measured in the whole blood by ELISA.

#### 2.2.4 Experiment 4: Effect of ovariectomy and estradiol replacement on LH and FSH secretion in rats determined by ultrasensitive ELISA

LH and FSH levels increase after ovariectomy in female rats, and estradiol treatment in OVX rats results in negative and positive feedback effects on LH secretion in the morning and afternoon, respectively.^31^, ^43^, ^44^, ^45^ This experiment aimed to characterize the patterns of LH and FSH secretion after ovariectomy and subsequent estradiol replacement in female rats, measured in the tail‐tip blood by ultrasensitive ELISA. Experiments had a longitudinal design in which the same rats were evaluated in three different hormonal conditions. To investigate the negative feedback effect of estradiol, three blood samples of 20 μL were withdrawn hourly from 08:00 to 10:00 h (*n* = 7). The measurements of the three blood samples were averaged per rat for each experimental condition. Accordingly, blood samples were initially collected from gonad‐intact rats on diestrus (Intact condition). Within 5 days, rats were OVX and, after 10 days of recovery, received corn oil (s.c.) for 3 consecutive days, and blood collection was performed on the next day (OVX condition). Fifteen days later, the OVX rats were then treated with estradiol for 3 consecutive days and, on the following day, blood samples were collected hourly from 8:00 to 10:00 h (OVX + E2 condition, negative feedback). To determine the positive feedback of estradiol, the same regimen of estradiol treatment was used, and additional nine blood samples were collected hourly from 11:00 to 19:00 h from a cohort of OVX + E2 rats (OVX + E2 condition, positive feedback; *n* = 5). LH and FSH were measured in the whole blood by ultrasensitive ELISA.

### 2.3 Surgical procedures and drug treatment

For ovariectomy or orchiectomy, rats were anesthetized with intraperitoneal (i.p.) ketamine (80 mg/kg body weight, b.w.) and xylazine (10 mg/kg b.w.). After surgeries, rats were treated with pentabiotic (24,000 UI/kg b.w., intramuscular; Fort Dodge, Campinas, Brazil) and analgesic (Flunixin meglumine, 2.5 mg/kg b.w., s.c.). Testosterone (0.25 mg/0.1 mL/rat, s.c.; testosterone propionate, Organon, Sao Paulo, Brazil) was diluted in corn oil with 10% ethanol and administered at 9:00 h. The regimen of testosterone treatment used yields physiological testosterone levels and restores LH levels and prostate weight in ORX rats.^46^, ^47^ Estradiol (80 μg/kg b.w., s.c.; 17β‐estradiol, Sigma‐Aldrich, St. Louis, MO, USA) was diluted in corn oil with 10% ethanol and administered daily for 3 days at 9:00 h. This regimen of estradiol treatment in OVX rats yields physiological levels of circulating estradiol, restores uterine weight, inhibits LH secretion in the morning, and generates a proestrus‐like surge of LH in the afternoon.^45^

### 2.4 Tail‐tip blood sampling

Blood collection from the rat tail tip was performed as previously described.^36^, ^37^ Rats were handled daily for at least 30 days prior to the experiment to acclimate them to the procedure of tail‐tip blood sampling. Immediately before bleeding, the distal 1–2 mm of the tail was clipped, and 20 μL tail‐tip blood samples were collected using a micropipette at 1‐h intervals at the time points predicted in each experiment. Whole blood samples were immediately suspended in phosphate‐buffered saline (PBS) with 0.05% Tween‐20 (PBS‐T) at a dilution of 1:20, placed on ice, and stored at −20°C until LH and FSH measurements.

### 2.5 ELISA

**FIGURE 1:**
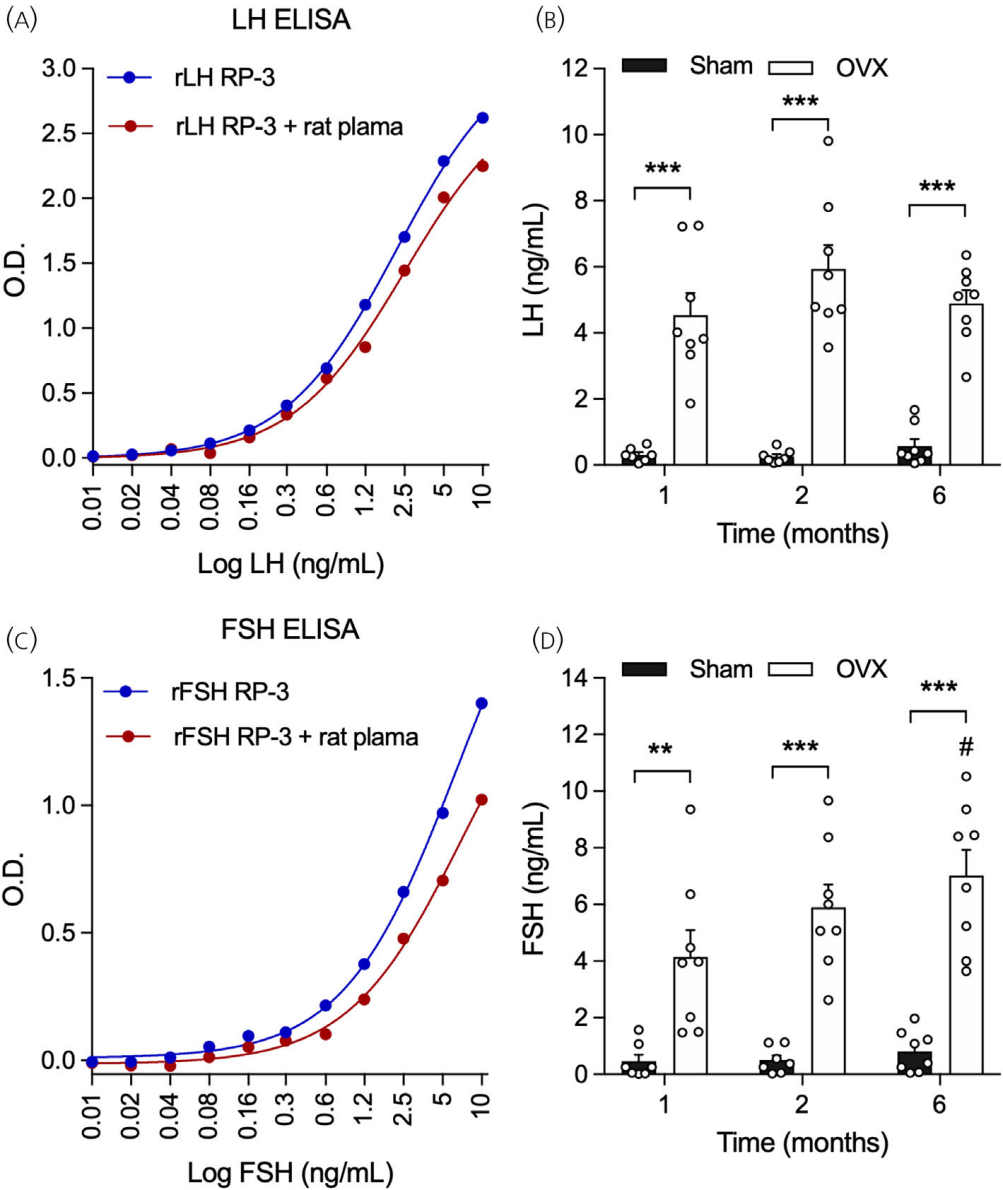
Luteinizing hormone (LH) and follicle‐stimulating hormone (FSH) ultrasensitive enzyme‐linked immunosorbent assays (ELISAs) reliably detect changes in gonadotropin secretion in rats. (A, C) Standard curves were generated using rat (A) LH and (C) FSH reference preparations in the absence (blue) or presence (red) of 20% rat plasma. (B, D) Sham‐operated (Sham) and ovariectomized (OVX) rats were euthanized at 1 (Sham‐1 M; *n* = 7; OVX‐1 M; *n* = 8), 2 (Sham‐2 M; *n* = 7; OVX‐2 M; *n* = 8), or 6 (Sham‐6 M; *n* = 8; OVX‐6 M; *n* = 8) months after the surgery. Plasma (B) LH and (D) FSH levels were measured by ELISA. Data shown as mean ± SEM. **p < .01, ***p < .001 Sham vs. OVX; ^#^ *p* < .05 OVX‐6 M vs. OVX‐1 M, determined by two‐way ANOVA followed by the Bonferroni post hoc test.

Rat plasma (experiment 1) and whole blood (experiments 2, 3, and 4) LH and FSH levels were assayed by ultrasensitive sandwich ELISA. The LH ELISA was adapted from a previously described method.^36^, ^37^ Briefly, a 96‐well high‐binding plate (9018, Corning, Kennebunk, ME, USA) was coated with 50 μL of the monoclonal anti‐bovine LH‐β antibody (518B7, UC Davis; RRID: AB_2756886) in PBS at 1:2500 dilution overnight at 4°C. The coating antibody was decanted, and wells were incubated with 200 μL of blocking buffer (5% skim milk powder in PSB‐T) for 2 h at room temperature (RT). A standard curve was prepared using a 2‐fold serial dilution of rat LH RP‐3 [AFP718B, National Institute of Diabetes and Digestive and Kidney Diseases—National Hormone and Pituitary Program (NIDDK‐NHPP)] in PBS‐T with 0.2% bovine serum albumin (BSA). Wells were incubated with 50 μL of samples at 1:20 dilution overnight at RT. The plate was washed, and wells were incubated with 50 μL of the rabbit anti‐rat LH antibody (AFP240580Rb, NIDDK‐NHPP; RRID: AB_2665533) in PBS‐T with 0.2% BSA at 1:100,000 for 6 h at RT. After washing, wells were incubated with 50 μL of horseradish peroxidase‐conjugated goat anti‐rabbit IgG (P044801‐2, Dako Pathology Solutions, Santa Clara, CA; RRID: AB_2617138) diluted in 50% PBS, 50% blocking buffer at 1:2000 for 90 min at RT. After a final wash, wells were incubated with 100 μL of 2‐mg/mL o‐phenylenediamine dihydrochloride (P1526, Sigma‐Aldrich, St. Louis, MO, USA) in citrate–phosphate buffer (pH 5.0) containing 0.02% hydrogen peroxide for 45 min at RT. The reaction was stopped with 50 μL of 3 M hydrochloric acid. The absorbance was then determined at 490 nm using a microplate reader, and a 650‐nm wavelength was used for background correction. The lower limit of detection was 0.070 ng/mL, and the intra‐assay and inter‐assay coefficients of variation were 3.3% and 10.4%, respectively. The rat FSH ELISA was developed using the same protocol as the LH ELISA described above, with the substitution of the specific antibodies and reference preparation. Accordingly, the 96‐well high‐binding plate was coated with 50 μL of the guinea pig anti‐rat FSH (anti‐rFSH beta‐IC‐3, AFP28122491, NIDDK‐NHPP; RRID: AB_3105815) in PBS at 1:2000 overnight at 4°C. A standard curve was prepared using a 2‐fold serial dilution of rat FSH RP‐2 (AFP4621B, NIDDK‐NHPP) in PBS‐T with 0.2% BSA. Wells were incubated with 50 μL of samples at 1:20 dilution overnight at RT. The plate was washed, and wells were incubated with 50 μL of the rabbit anti‐rat FSH antibody (anti‐rFSH beta‐IC‐2, AFPHFSH6, NIDDK‐NHPP; RRID: AB_3713254) in PBS‐T with 0.2% BSA at 1:10,000 for 6 h at RT. Wells were then incubated with the horseradish peroxidase‐conjugated goat anti‐rabbit IgG (P044801‐2, Dako Pathology Solutions, RRID: AB_2617138) and o‐phenylenediamine dihydrochloride (P1526, Sigma‐Aldrich). The absorbance was determined at 490 nm, and a reading at 650 nm was used for background correction. The lower limit of detection was 0.154 ng/mL, and the intra‐assay and inter‐assay coefficients of variation were 5.4% and 9.9%, respectively. All samples from the same experiment were assayed in a single assay. LH and FSH levels were obtained by interpolating the optical density of the samples against a non‐linear regression of the respective standard curves. Parallelism tests were performed generating LH and FSH standard curves diluted in PBS‐T with 0.2% BSA in the absence or presence of 20% rat plasma (gonad‐intact male rat; Figure 1A,C).

### 2.6 Statistical analysis

**FIGURE 2:**
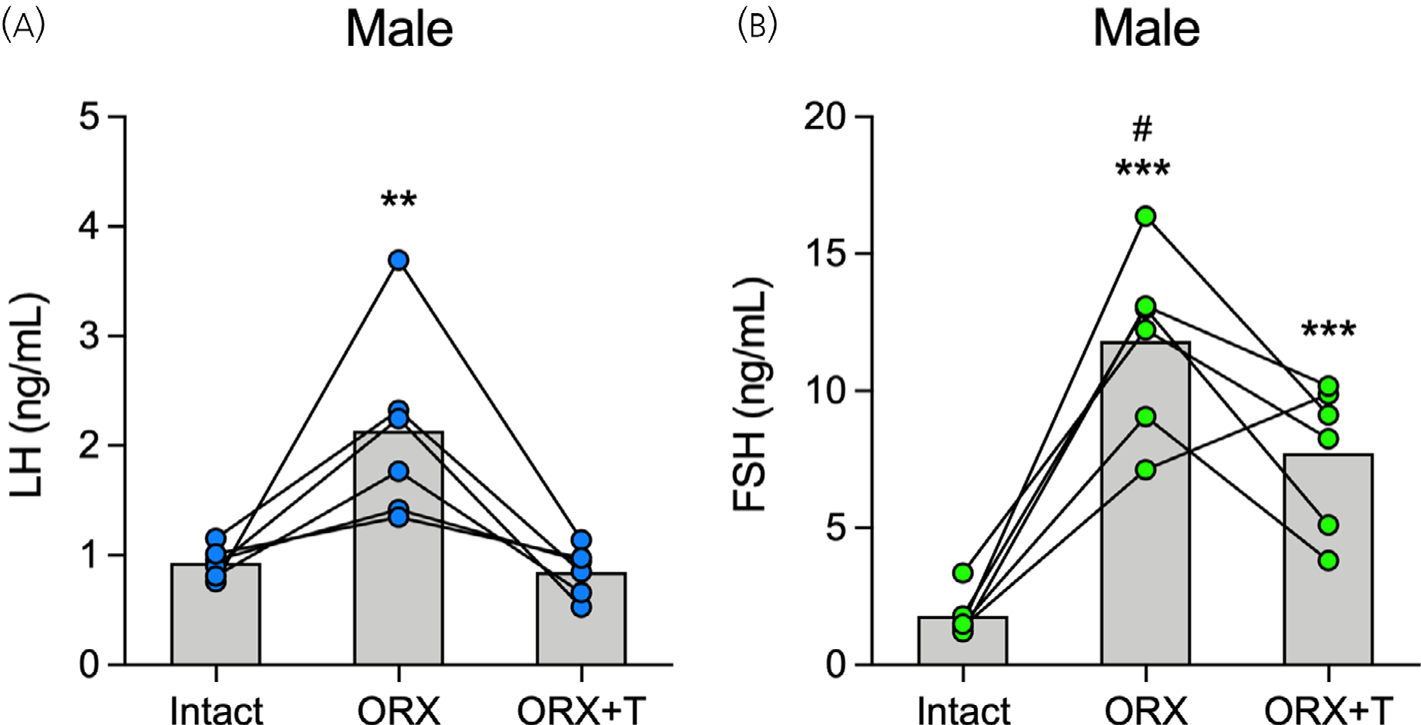
Effects of orchiectomy and testosterone replacement on luteinizing hormone (LH) and follicle‐stimulating hormone (FSH) secretion in rats measured by enzyme‐linked immunosorbent assay (ELISA). Male rats were longitudinally studied at three subsequent time points under different hormonal conditions (*n* = 6): Gonad‐intact (Intact), orchiectomized (ORX) treated with corn oil, and ORX treated with testosterone (ORX + T). Three tail‐tip blood samples of 20 μL were withdrawn hourly from 10:00 to 12:00 h in each condition, and LH and FSH levels were measured in whole blood using ELISA. The measurements of three blood samples were averaged per rat for each experimental condition. Mean and individual data points of (A) LH and (B) FSH levels are shown. Lines connecting the dots represent the same animal across the three hormonal conditions. LH: **p < .01 compared with Intact and ORX + T. FSH: ***p < .001 compared with Intact; ^#^ *p* < .05 compared with Intact and ORX + T. Differences were determined by one‐way ANOVA for repeated measures followed by the Bonferroni post hoc test.

**FIGURE 3:**
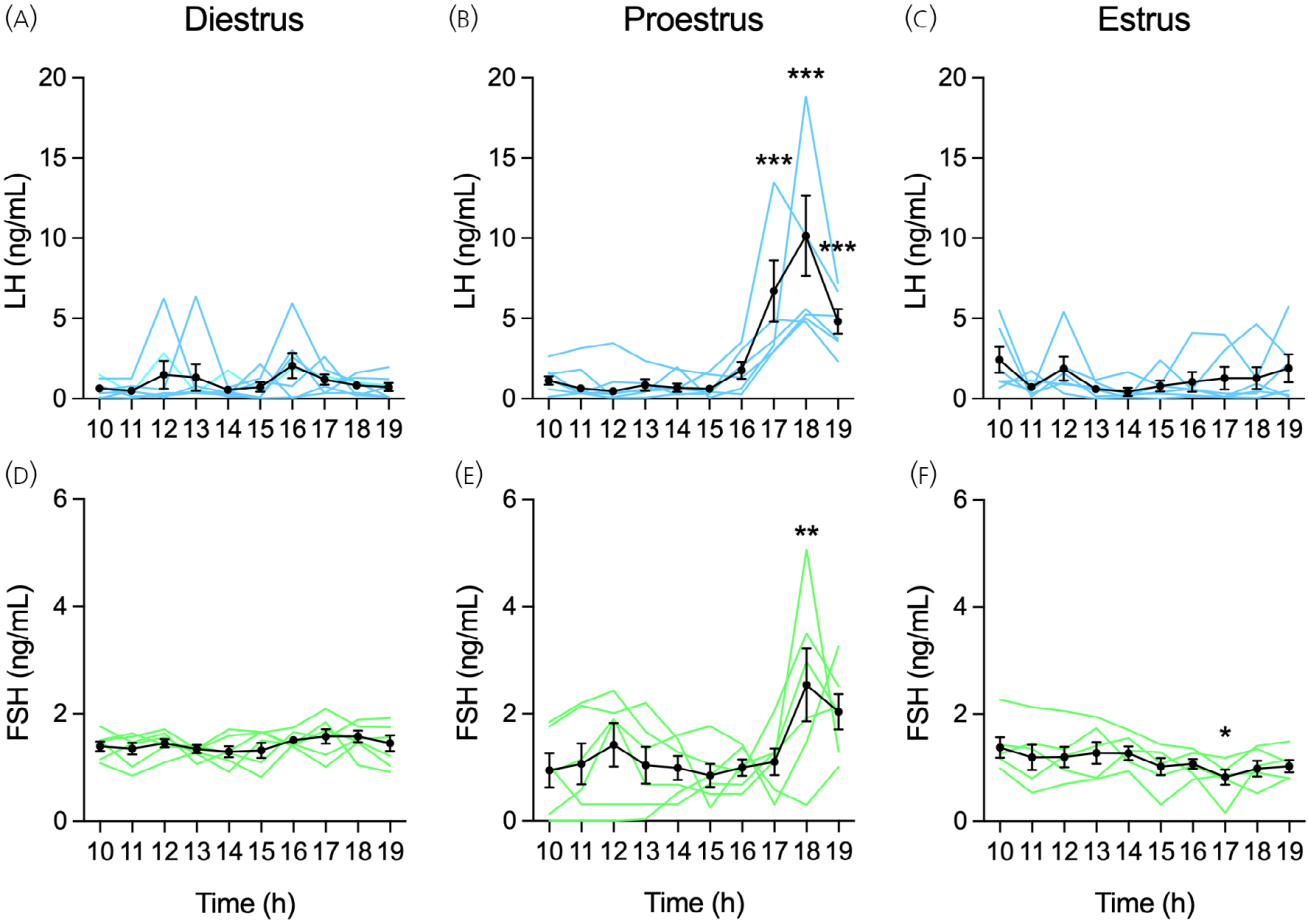
Luteinizing hormone (LH) and follicle‐stimulating hormone (FSH) secretion during the rat estrous cycle measured by enzyme‐linked immunosorbent assay (ELISA). Tail‐tip blood samples were withdrawn hourly from 10:00 to 19:00 h from cohorts of rats on diestrus (*n* = 7), proestrus (*n* = 6), or estrus (*n* = 6). LH and FSH were measured in whole blood using ELISA. (A–F) Mean ± SEM and individual temporal profiles of LH and FSH levels in female rats on (A, D) diestrus, (B, E) proestrus, and (C, F) estrus. *p < .05, **p < .01, ***p < .001 compared with 10:00 h on the same day, determined by one‐way ANOVA for repeated measures followed by the Bonferroni *post‐hoc* test.

**FIGURE 4:**
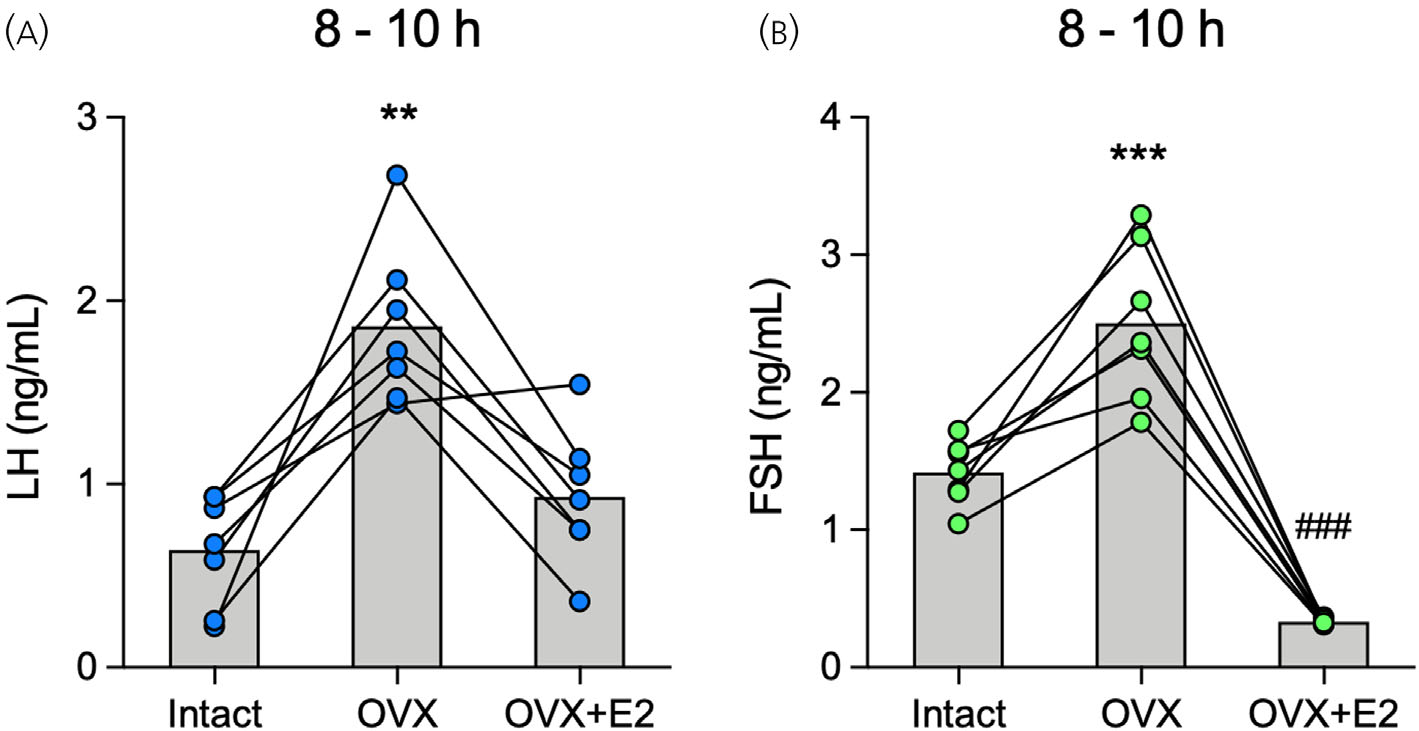
Effects of ovariectomy and estradiol negative feedback on luteinizing hormone (LH) and follicle‐stimulating hormone (FSH) secretion in rats measured by enzyme‐linked immunosorbent assay (ELISA). Female rats were longitudinally investigated in three subsequent time points under different hormonal conditions (*n* = 7): Gonad‐intact on diestrus (Intact), ovariectomized (OVX) treated with corn oil, and OVX treated with estradiol (OVX + E2). Three tail‐tip blood samples of 20 μL were withdrawn hourly from 8:00 to 10:00 h in each condition, and LH and FSH levels were measured in whole blood using ELISA. The measurements of the three blood samples were averaged per rat for each experimental condition. Mean and individual data points of (A) LH and (B) FSH levels. Lines connecting the dots represent the same animal across the three hormonal conditions. LH: **p < .01 compared with Intact and OVX + E2. FSH: ***p < .001 compared with Intact and OVX + E2; ^###^ *p* < .001 compared with Intact. Differences were determined by one‐way ANOVA for repeated measures followed by the Bonferroni post hoc test.

Data are presented as mean ± standard error of the mean (SEM) and analyzed with GraphPad Prism (GraphPad Software, San Diego, USA). Data were checked for normality and homogeneity of variance before performing comparisons by analysis of variance (ANOVA). Two‐way ANOVA followed by the Bonferroni *post‐hoc* test (experiment 1) or one‐way ANOVA for repeated measures followed by the Bonferroni *post‐hoc* test (experiments 2, 3, and 4). LH and FSH data in male rats (Figure 2), LH data on proestrus (Figure 3B), and FSH data in female rats at 8–10 h (Figure 4B) were logarithmically transformed to meet the criteria of homogeneity of variance before one‐way ANOVA. *p* < .05 was considered statistically significant.

## 3 RESULTS

### 3.1 Validation of rat LH and FSH ultrasensitive ELISAs

Figure 1 shows standard curves of rat LH (Figure 1A) and FSH (Figure 1C) ultrasensitive ELISAs, generated using the respective hormone reference preparations diluted with or without the addition of rat plasma to test for its interference on the assays. The standard curves show that detection of LH and FSH was minimally affected by the presence of rat plasma. In both assays, a more noticeable negative influence of rat plasma was found at the level of the highest reference preparation concentrations (10 ng/mL). However, virtually no plasma or blood sample is quantified at this region of the curve, indicating that LH and FSH detections were not significantly affected by non‐specific interactions with rat proteins. Additionally, the LH and FSH ELISAs were effective in detecting the increase in gonadotropin levels in the plasma of female rats subjected to short‐ and long‐term ovariectomy. As determined by two‐way ANOVA, there was a main effect of ovariectomy on plasma LH levels (*F*_1,40_ = 154.9, *p* < .001) but not of time or interaction between factors. Accordingly, LH levels were similarly increased in OVX rats compared with Sham at all time points evaluated after ovariectomy (*p* < .001; Figure 1B). Likewise, there was a main effect of ovariectomy on plasma FSH (*F*_1,40_ = 85.10, *p* < .001) with no effect of time or interaction between factors. As determined by the Bonferroni *post‐hoc* test, OVX rats displayed higher FSH secretion than Sham rats at 1, 2, and 6 months after ovariectomy (*p* < .01), with a further increase in FSH secretion detected in OVX‐6 M rats compared with the OVX‐1 M ones (*p* < .05; Figure 1D). Altogether, these results demonstrate that the LH and FSH ultrasensitive ELISAs reliably detect expected changes in gonadotropin secretion in rats.

### 3.2 Effect of orchiectomy and testosterone replacement on LH and FSH secretion in rats determined by ultrasensitive ELISA

We then determined the effects of orchiectomy and testosterone treatment on LH and FSH secretion, which were longitudinally measured in whole blood using ultrasensitive ELISAs (Figure 2). As expected, blood LH levels were altered by orchiectomy and testosterone replacement (*F*_2,10_ = 16.64, *p* < .001). Orchiectomy increased LH levels compared with the Intact condition (*p* < .01). The following testosterone replacement reduced LH secretion in the ORX + T condition compared with ORX (*p* < .01), restoring it to the basal levels of the Intact condition (Figure 2A). Similar to the LH response, FSH secretion was also altered by orchiectomy and testosterone treatment (*F*_2,10_ = 50.65, *p* < .001). Blood FSH levels markedly increased in the ORX condition compared with the Intact (*p* < .001). After testosterone treatment, FSH levels were significantly reduced in the ORX + T condition compared with ORX (*p* < .05). However, FSH levels in the ORX + T condition remained significantly higher than the basal levels of the Intact (*p* < .001; Figure 2B), demonstrating that, unlike LH, testosterone treatment did not fully restore the gonad‐intact levels of FSH in male rats. The prostate weight in the ORX + T condition (81.0 ± 4.3 mg/100 g b.w.; *n* = 6) was consistent with that found in gonad‐intact rats,^47^ confirming the efficacy of testosterone replacement in the physiological range in this experiment.

### 3.3 LH and FSH secretion during the rat estrous cycle determined by ultrasensitive ELISA

In this experiment, we determined the fluctuations of LH and FSH levels in the whole blood across the rat estrous cycle (Figure 3). On diestrus, LH levels were low with discrete individual oscillations but no significant change during the day (*F*_9,54_ = 1.12, *p* = .36; Figure 3A). On proestrus, LH levels remained at baseline values with less individual oscillations in the morning. The afternoon was marked by the preovulatory LH surge, in which LH levels on average began to rise about 16:00 h, reached peak levels at 18:00 h, and started to decline at 19:00 h (*F*_9,45_ = 12.52, *p* < .001; Figure 3B). On estrus, there was a substantial reduction in blood LH levels, which despite individual oscillations remained at unchanged basal levels throughout the day (*F*_9,45_ = 0.21, *p* = .21; Figure 3C). As for FSH secretion, on diestrus, blood levels of this hormone displayed low variability and remained constantly low throughout the day (*F*_9,54_ = 1.15, *p* = .34; Figure 3D). During proestrus, FSH levels showed increased variability but with no significant change until 17:00 h, when FSH levels on average increased and reached the peak of the preovulatory surge at 18:00 h (*F*_9,45_ = 3.25, *p* < .01; Figure 3E). FSH levels were undetectable in three samples of one rat from 10:00 to 12:00 h on proestrus. On estrus, FSH secretion slowly declined from 10:00 h to the lowest concentration at 17:00 h (*F*_9,45_ = 2.12, *p* < .05; Figure 3F).

### 3.4 Effect of ovariectomy and estradiol replacement on LH and FSH secretion in rats determined by ultrasensitive ELISA

**FIGURE 5:**
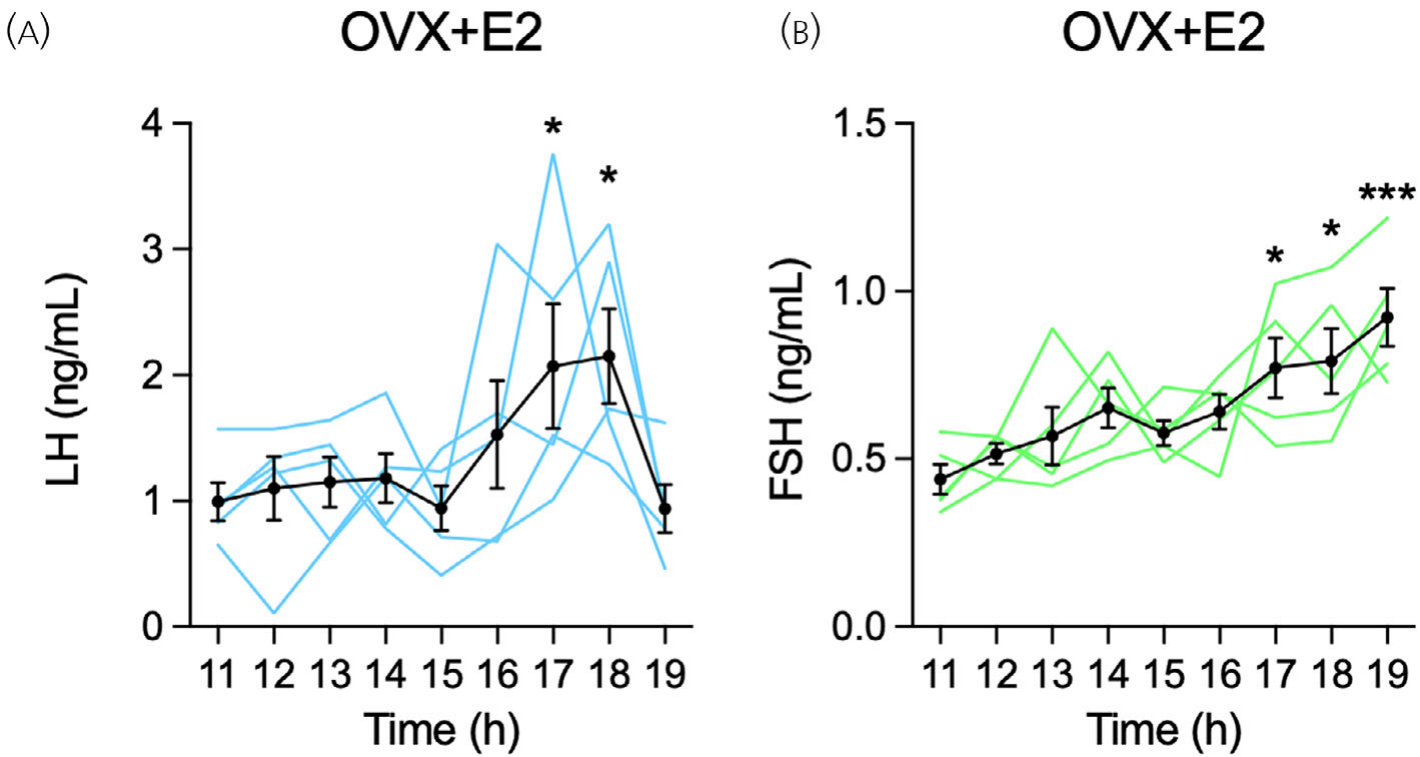
Positive‐feedback effects of estradiol on luteinizing hormone (LH) and follicle‐stimulating hormone (FSH) secretion in ovariectomized (OVX) rats measured by enzyme‐linked immunosorbent assay (ELISA). Female rats were longitudinally studied in three subsequent time points under different hormonal conditions: Diestrus, OVX, and OVX treated with estradiol (OVX + E2). In the OVX + E2 model, OVX rats were treated with estradiol for 3 consecutive days. On the fourth day, tail‐tip blood samples of 20 μL were collected hourly from 11:00 to 19:00 h, and whole blood LH and FSH levels were measured using ELISA. Mean ± SEM and individual temporal profile of (A) LH and (B) FSH levels in OVX + E2 rats (*n* = 5). *p < .05, ***p < .001 compared with 11:00 h, determined by one‐way ANOVA for repeated measures followed by the Bonferroni post hoc test.

Next, we evaluated the effects of ovariectomy and the bimodal outcome of estradiol replacement on gonadotropin secretion in rats, longitudinally measured in whole blood using ultrasensitive ELISAs (Figures 4 and 5). Estradiol is known to exert a dual action on gonadotropin secretion in OVX rats. This action includes the inhibition of LH and FSH secretion in the morning, as a result of the negative‐feedback effect, and the stimulation of an afternoon LH surge by the positive‐feedback.^29^, ^30^, ^43^, ^45^ Between 8:00 and 10:00 h, as expected, blood LH levels in the OVX condition were higher compared with Intact, and the estradiol treatment in the OVX + E2 condition fully restored LH levels to the gonad‐intact values (*F*_2,12_ = 21.90, *p* < .001; Figure 4A). Likewise, during the morning, FSH secretion in OVX was higher than Intact and OVX + E2 conditions (*F*_2,12_ = 341.5, *p* < .001). Estradiol treatment, in turn, not only restored FSH secretion, but further decreased it to lower levels than in the Intact mode (*p* < .001; Figure 4B). We then evaluated the profile of gonadotropin secretion from 11:00 to 19:00 h in the OVX + E2 condition to assess the positive‐feedback effect of estradiol. Accordingly, blood LH concentrations were low until 15:00 h, when the estradiol‐induced LH surge started with rising levels of LH around 16:00 h, peak levels between 17:00 and 18:00 h, and return to basal levels at 19:00 h (*F*_8,32_ = 3.50, *p* < .01; Figure 5A). Estradiol also elicited an afternoon increase in FSH secretion. However, unlike the LH surge, the estradiol‐induced rise in FSH was characterized by a continuous, gradual increase in this hormone secretion, which reached higher blood levels from 17:00 to 19:00 h compared with the morning period (*F*_8,32_ = 4.53, *p* < .001; Figure 5B). Confirming the efficacy of the estradiol treatment, uterine weight in the OVX + E2 condition (399.8 ± 45.8 mg/100 g b.w.; *n* = 7) was consistent with that previously found on proestrus and in OVX + E2 rats under the same estradiol treatment regimen.^45^

## 4 DISCUSSION

In the present study, we used highly sensitive ELISAs to measure LH and FSH levels in micro samples of whole blood in rats under different hormonal conditions in order to characterize the gonadotropin secretion in this species as determined by this new methodology. We initially validated the accuracy of the ELISAs in detecting rat LH and FSH levels with the addition of rat plasma and their efficacy in detecting the expected rise in gonadotropins after ovariectomy. Subsequently, we longitudinally characterized LH and FSH secretion in the tail‐tip blood of male and female rats under different regulation by gonadal steroids. The expected variation in gonadotropin and novel information regarding the estradiol‐induced FSH surge are reported in measurements performed in micro‐volume blood samples (50 μL at 1:20 dilution) serially obtained from vein cannulation‐free unstressed rats, providing relevant methodological and physiological information about gonadotropin secretion in rats that is relevant to the knowledge in reproductive endocrinology.

The LH and FSH ELISAs were sensitive in detecting alterations in the secretion of these gonadotropins in Sham‐operated and OVX rats at different time points after surgery. Plasma LH and FSH levels were significantly elevated in OVX rats compared to Sham at all time points investigated after ovariectomy, as expected by the removal of ovarian‐steroid negative feedback on gonadotropin secretion.^48^, ^49^ Furthermore, we observed that longer periods of time after ovariectomy do not influence LH secretion, which was equally elevated from 1 to 6 months after surgery (rats with 4 to 9 months of age). On the other hand, FSH secretion displayed a gradual rise over time in OVX rats, with higher FSH levels at 6 months compared to 1 month after ovariectomy. These results demonstrate that, in the absence of ovarian steroids, FSH levels rise gradually over time, whereas LH secretion reaches a plateau of maximum secretion by 1 month. The effect of short‐term gonadectomy on gonadotropin secretion has been demonstrated in rats by RIA.^49^, ^50^ Our results are consistent with the previous reports and also suggest that the patterns of LH and FSH secretion differ in long‐term ovariectomy in rodents, although the neuroendocrine mechanisms underlying these differential temporal responses remain to be determined.

Gonadectomy in male and female rats increases LH and FSH secretion by interrupting the negative‐feedback effect mediated by sex steroids and inhibins, and it has been demonstrated that treatments with testosterone and estradiol in gonadectomized rats reduce LH and FSH secretion, subject to the influence of the hormone and regimen of treatment used.^41^, ^42^, ^51^, ^52^, ^53^, ^54^, ^55^ In the present study, blood LH and FSH levels were elevated in ORX compared to Intact rats. The testosterone replacement in physiological levels^46^, ^47^ restored LH secretion to the baseline values of the gonad‐intact animals. FSH was also reduced by testosterone, but unlike LH, its secretion in ORX + T rats was not completely restored to the gonad‐intact levels. These results demonstrate that LH is more sensitive than FSH to testosterone inhibition, consistent with previous evidence obtained using other methodologies. In this line, early RIA studies indicated that higher doses of testosterone were required to suppress serum FSH levels to the same extent as LH in adult ORX rats.^41^ In turn, a recent study employing commercial ELISA reported that supra‐physiological dihydrotestosterone (3 mg/kg; yielding a 2‐fold greater prostate weight than in intact animals) administered to ORX rats was able to equally suppress LH and FSH secretion.^56^ These reports are consistent with our ELISA data showing that testosterone replacement in ORX rats fully restores negative feedback on LH but not FSH secretion. These data reflect differences in the neuroendocrine control of LH and FSH secretion in male rats.

The secretion of LH and FSH throughout the rat estrous cycle and the preovulatory surge of LH have been characterized by earlier studies using RIA.^10^, ^26^, ^27^, ^28^, ^29^, ^57^ Although ultrasensitive ELISAs have been used to evaluate the gonadotropin levels in the blood of mice and rats,^32^, ^33^, ^34^, ^36^, ^37^, ^58^ this methodology has not been employed to measure LH and FSH secretion across the rat estrous cycle. The ELISA measurements in serial tail‐tip blood provided an accurate profile of gonadotropin secretion during the estrous cycle, consistent with the earlier reports determined by RIA in the trunk blood of decapitated rats. LH and FSH displayed basal levels on estrus and diestrus, and the preovulatory surges of these gonadotropins happened on the afternoon of proestrus. The rise in blood levels during the proestrous surge was greater for LH than for FSH. In turn, FSH secretion showed a gradual, slower decay on estrus. The LH and FSH peaks on proestrus were approximately 10 and 3 times higher than the basal levels, respectively. Earlier RIA studies reported increases of approximately 15 to 150 times for LH and 3 to 5 times for FSH during the proestrous surges.^10^, ^26^, ^27^, ^28^, ^29^, ^57^ Thus, despite the differences in experimental methodologies, the magnitude of the proestrous surges of LH and FSH reported here is consistent with previous RIA measurements. The preovulatory surge of gonadotropins is induced by the rise in estradiol levels from diestrus to proestrus.^10^, ^26^ Accordingly, the immunoneutralization of estradiol blocks the LH surge on proestrus, while estradiol treatment in OVX rats generates an afternoon LH surge.^29^, ^30^, ^43^, ^45^ Although a similar estradiol regulation is expected for FSH, there is only sparse information on the occurrence of an estradiol‐induced FSH surge in OVX rats.^30^, ^59^ In addition, the secretion of LH showed an apparent higher individual variability than that of FSH across the estrous cycle. Of note, the moment of lowest individual variability for LH was the morning of proestrus, whereas for FSH it was on diestrus. These differences seem to reflect distinct neuroendocrine controls, including a differential responsivity of LH and FSH to GnRH.^53^, ^60^, ^61^

We further evaluated the longitudinal effects of ovariectomy and estradiol replacement on LH and FSH secretion in the same rats. During the morning period, as expected from the removal of the negative feedback signals from the ovary, ovariectomy similarly increased blood LH and FSH compared to the ovary‐intact condition, and both hormones were reduced to basal levels by physiological estradiol replacement.^45^ These results are consistent with earlier RIA studies showing that estradiol negative feedbacks to inhibit LH and FSH secretion in the morning in OVX rats.^30^, ^43^ Notably, unlike LH, FSH secretion in the OVX + E2 condition was further reduced by estradiol to lower levels than in the ovary‐intact condition. As FSH and LH were measured longitudinally in the same cohort of rats, these data suggest that FSH is more sensitive than LH to the negative‐feedback effect of estradiol in female rats.

The afternoon surges of LH and FSH induced by estradiol in OVX + E2 rats were accurately demonstrated by the ELISA measurement in the tail‐tip blood. LH secretion displayed a sharp surge in the late afternoon, followed by a return to baseline levels. FSH secretion, in turn, slowly increased during the afternoon and early evening. Although in lower magnitude, the temporal profiles of the estradiol‐induced rise in gonadotropins reflect those of the preovulatory surges on proestrus, depicting a sharp and prominent LH peak, as opposed to a slow and gradual rise in FSH levels. The estradiol‐induced LH surge reported here is consistent with previous reports using RIA^30^, ^43^, ^45^ and ELISA.^36^, ^45^ Notably, the size of the LH surge was smaller in OVX + E2 rats as compared to proestrus, which is explained by the facilitatory role of progesterone, present on proestrus but not in the OVX + E2 model. Progesterone is known to further activate the GnRH neurons and amplify the estradiol‐induced LH surge.^59^, ^62^, ^63^, ^64^ On the other hand, little is known about the FSH surge induced by estradiol. Using RIA, Caligaris et al.^30^ reported a slight, non‐significant increase in afternoon FSH levels in OVX + E2 rats, determined in a single time‐point measurement. Investigating different days of estradiol priming in OVX + E2 rats, DePaolo and Barraclough^59^ found an afternoon increase in plasma FSH after 4, but not 3 or 5, days of treatment. These authors also reported that, when treated with progesterone, OVX + E2 rats primed with estradiol for 3 days were able to mount an FSH surge. Thus, our data describes the profile of the estradiol‐induced FSH surge in rats. The comparison between proestrus and OVX + E2 rats suggests that other factors, such as progesterone, should play a role alongside estradiol in the proestrous FSH surge.

Herein, we adapted the FSH ELISA for the rat and characterized LH and FSH secretion in male and female rats under different hormonal conditions, measured by ELISA in the tail‐tip blood. While the gonadotropin profiles have been determined by earlier studies using RIA, this is the first comprehensive description involving longitudinal measurements in rats using the ultrasensitive ELISA. Although not performed here, the proportionality between the ELISA and RIA for LH and FSH has been previously reported.^32^, ^33^ Notably, these ELISAs use NIDDK‐NHPP antibodies, which have unfortunately been discontinued. Nevertheless, these assays can work with other antibodies, as has been shown for the LH ELISA.^65^ Upon development and testing of new antibodies, the ultrasensitive ELISA is likely to continue to be used as an important hormonal assay. Thus, our present findings provide relevant methodological and physiological information about gonadotropin secretion in rats, which adds to the current knowledge and will contribute to future studies in the field of research.

## FUNDING INFORMATION

This study was supported by Conselho Nacional de Desenvolvimento Cientifico e Tecnologico (CNPq; to Soraia Macari and Raphael E. Szawka), Fundacao de Amparo a Pesquisa do Estado de Minas Gerais (FAPEMIG; to Andre F. Gomes, Adelina M. Reis, and Raphael E. Szawka), Coordenacao de Aperfeicoamento de Pessoal de Nivel Superior (CAPES; to Roberta Araujo‐Lopes).

## CONFLICT OF INTEREST STATEMENT

The authors declare no conflict of interest.

## Data Availability

The data that support the findings of this study are available from the corresponding author upon reasonable request.
